# Neuromuscular Fitness Is Associated with Serve Speed in Young Female Tennis Players

**DOI:** 10.3390/sports12040097

**Published:** 2024-03-30

**Authors:** Zlatan Bilić, Paola Martić, Petar Barbaros, Filip Sinković, Dario Novak

**Affiliations:** Faculty of Kinesiology, University of Zagreb, 10000 Zagreb, Croatia; zlatan.bilic@kif.unizg.hr (Z.B.); paola.martic@student.kif.unizg.hr (P.M.); petar.barbaros@kif.unizg.hr (P.B.); filip.sinkovic@kif.unizg.hr (F.S.)

**Keywords:** serve speed, upper extremities, strength interaction, predictive model

## Abstract

In tennis, the serve plays a key role in determining the success of a player. The speed of a serve is influenced by a multitude of interconnected skills and abilities. The objective of this study was to establish the correlation between the explosive strength of the throwing type, the grip strength and flexibility of the arms, and the shoulder girdle with the serve speed in young female tennis players. Additionally, the study aimed to develop a regression model that accurately predicts the serve speed by analyzing the interplay among these variables. The study was carried out on a group of 20 tennis players, who had an average age of 13.10 ± 0.74 years. Additionally, their height was recorded as 165.70 ± 4.90 cm, and their body mass was measured at 51.45 ± 5.84 kg. To assess the motor abilities of the upper extremities, four tests were used that aimed to measure the explosive strength of the throwing type; one test was for the strength of the hand and forearm muscles, and one test was for the flexibility of the arms and shoulder girdle. Of all the variables examined, the medicine ball throw shot put (MBTSP) (r = 0.75), overhead medicine ball throw (OMBT) (r = 0.70), and grip strength (GS) (r = 0.71) displayed a notable correlation with serve speed (*p* < 0.05). The results obtained from the multiple regression analysis indicate that the combination of selected predictors (MBTSP—medicine ball throw shot put, OMBT—overhead medicine ball throw and GS—grip strength) explained 75% of the variability in serve speed. Significantly, MBTSP surfaced as the predominant predictor, autonomously elucidating 51% of the variability in serve speed. The importance of improving the analyzed motor skills of young female tennis players to enhance their serve in terms of speed is emphasized by the findings of this research.

## 1. Introduction

In modern tennis, the serve is considered as one of the most dominant shots in the game, as well as one of the most important shots from a strategic point of view too [1]. The serve is unique because it allows the player to have a greater degree of control over the shot as it is less influenced by the opponent. This specific characteristic of the serve gives tennis players more freedom in performing the shot and allows them to achieve greater precision and speed. The effective performance of serves in tennis requires the proper activation and coordination of different parts of the body via the concept of the kinetic chain. The complexity of serving movements stems from the combination of limb and joint movements needed to sum up and transfer forces from the ground, through the kinetic chain, to the ball [1]. The activation and coordination of multiple segments within the kinetic chain, such as the lower extremities, trunk, and upper extremities, are widely acknowledged as essential for achieving optimal force [2]. If any of these elements are not effectively synchronized, the serve outcome will not be optimal. The success of a serve is influenced by various crucial factors that significantly contribute to its effectiveness. The elements that contribute to the serve’s effectiveness encompass the speed, the angle of impact, the spin imparted on the ball, and the level of precision [3,4]. The high speed of the serve is a crucial element that has a notable impact on the results of service games in men’s and women’s tennis, as well as in junior players [5,6]. Players who possess the skill to execute a high serve speed can launch a powerful attack, apply pressure on their opponents, and reduce their reaction time, resulting in difficulties for their opponents to return the serve as intended. The efficient production of high-speed shots, particularly on the serve, is a crucial factor in the success of tennis players [7]. A significant correlation between the match-winning probability and the serve speed has been established in a previous study [8]. Furthermore, additional research [9] has discovered that the correlation between the percentage of points won on first serve and competitive success is substantial across various playing court surfaces.

The primary focus of this research revolves around biomechanical factors that impact the effectiveness of the serve. These factors include analyzing the angular torque values of specific body segments, the speed of the ball, and identifying the variables that influence the speed of the racket head [10]. Even though the biomechanical analysis of the tennis serve reveals a nearly equal distribution of kinetic energy generation between the upper and lower extremities, numerous players fail to maximize their serve speeds due to insufficient energy transfer from the lower extremities to the upper extremities. Consequently, they overly rely on the strength of their upper extremities [11]. Furthermore, numerous studies [12,13] have explored potential correlations between serve speed and various factors, such as anthropometric characteristics, player quality, flexibility, and strength through isokinetic and isometric testing. It is worth noting that the increased serve speed can be attributed to the greater muscle forces generated by the thrust of the lower extremities. In a recent study [14], it was found that there is a positive correlation between enhanced serve speed and greater body height among professional and junior players. Moreover, a previous study [5] has uncovered noteworthy correlations between serve speed and arm length in all investigated age groups. The speed of the serve is primarily linked to the players’ rankings across all age groups, underscoring the significance of ball speed in tennis proficiency. Additionally, it highlights the crucial role of early engagement in tennis and the intensity of training as influential factors that can greatly impact the enhancement of serve speed [15]. The increased range of motion (ROM) of the joints that are crucial for the kinetic chain of the serve seems essential for increasing speed production, so it turns out that internal shoulder rotation, wrist flexion, and external hip rotation positively affect the speed of serve, which confirms the importance of achieving adequate mobility in these joints to improve this component of the serve [16]. In addition, upper and lower limb strength measurements show uneven results, with moderate to no significant correlations with serve speed in junior and professional players when using isokinetic or isometric testing [17,18]. Given the complexity of the movement, the increase in serve speed is likely the result of a combination of multiple factors, including the player’s skills, anthropometry, and physical abilities. 

Previous studies in the realm of analyzing the elements that influence the speed of a tennis serve have concentrated on different facets of players’ physical abilities. These studies include the explosive strength of the throwing type, the strength of the fist grip, and the flexibility of the shoulder muscles. These factors have been identified as potential crucial factors in achieving a successful serve [19]. Previous similar research has primarily focused on professional and male players. There is a limited understanding of the specific motor skills that have the strongest correlation with the speed of serve in young female players. The identification of crucial elements that influence the speed of serve among female tennis players can greatly contribute to the enhancement and advancement of specific training programs designed to improve their serving skills. 

Due to all the aforementioned reasons, it is crucial to conduct testing using a sample that aligns with the parameters of this study. The objective of this research was to examine the correlation of the explosive strength of the throwing type, the grip strength and the shoulder girdle flexibility with the serve speed in female tennis players. Additionally, the study aimed to establish and refine a regression model that accurately predicts the speed of serve by analyzing the interplay among these variables.

## 2. Materials and Methods

### 2.1. Participants

The study included a group of 20 female tennis players, with an average age of 13.10 ± 0.74 years, height of 165.70 ± 4.90 cm, and body mass of 51.45 ± 5.84 kg. These players were among the top 40 ranked players in the National Tennis Federation rankings and practiced 5–6 days per week. Within the sample, 17 participants were right-handed, 3 were left-handed, and all of them had a two-handed backhand. To determine the required number of participants, the G-power program (version 3.1.9.4; Heinrich Heine University, Dusseldorf, Germany) was used, considering an expected effect power of f = 0.33, an alpha level of 0.05, and a statistical power of 0.90. Inclusion criteria included being in good shape, being a physically active player who trains at least 10 h per week and competing in national and international tournaments. All participants had to be healthy with no history of upper limb surgery, no pain in the shoulder, back, or knee in the past 6 months, and no rehabilitation in the past 6 months. Before conducting the test, the overall test procedure was carefully explained to all participants, as well as all the benefits and possible risks associated with this testing. The parents of the players also granted written permission for their child to take part in the research. Additionally, they were instructed to abstain from engaging in strenuous activities prior to the testing to prevent fatigue and physical strain from affecting the test outcomes. A comprehensive explanation of the testing procedure was provided. The study adhered to the 1964 Helsinki Declaration and its subsequent revisions, and it received approval from the Institutional Review Board of the Faculty of Kinesiology at the University of Zagreb (protocol code 12; approval date: 22 February 2023).

### 2.2. Instruments

To evaluate the explosive strength of the throwing type, a measuring tape of 15 m in length was used, along with medicine balls weighing 3 kg. To assess the strength of the hand and forearm muscles, a handheld dynamometer manufactured by Takei A5401 in Tokyo, Japan was used. To evaluate the range of motion in the arms and shoulder girdle, a wooden rod measuring 2.5 cm in diameter and 165 cm in length was used. A reference point, denoted as zero, was marked 15 cm from one end of the rod, and a centimeter scale was drawn from this point to the opposite end. The velocity of the service was determined using the Stalker ATS II radar, manufactured by Stalker Sport in Texas, USA.

### 2.3. Variables

An analysis was conducted to evaluate the motor skills, specifically focusing on the upper body, and to assess the explosive strength of the throw type. Four tests (OMBT—overhead medicine ball throw; BMBT—backward overhead medicine ball throw; MBTSP—medicine ball throw shot put; SMBT—seated medicine ball throw) were utilized for this purpose. Additionally, a grip strength test (GS) was used to evaluate the strength of the hand and forearm muscles, while twist with the stick test (TS) was used to assess the flexibility of the arms and shoulder girdle. Following the completion of these tests, an analysis was performed to determine the correlation between these assessments and the speed of serve (SS).

OMBT: The participant takes a standing position, with their feet positioned at the width of their hips. They place their fingers on the line mark on the floor and proceed to lift the medicine ball above their head using both hands. Before releasing the ball, a forceful swing is executed behind the head, followed by a forward-directed drop. It is important to note that any movement of the feet or stepping forward is prohibited. The throwing action is repeated twice, with a one-minute interval between each throw. During data analysis, only the best result out of the two throws is taken into consideration. The distance is measured in meters, from the line mark on the floor to the point of initial contact between the medicine ball and the surface.

BMBT: The participant positions himself with his heels aligned to the line mark, keeping his legs shoulder-width apart, and facing away from the throwing direction. He holds a 2 kg medicine ball above his head using both hands. The medicine ball is then brought down to knee level before being forcefully thrown, causing a rapid extension of the hips, knees, and ankles. The medicine ball is released backward, over the head. This throwing sequence is repeated twice, with a one-minute interval between throws. During further data analysis, a better result is considered when compared to the two throws that were performed.

MBTSP: The participant holds a 2 kg medicine ball in the palm of their dominant hand and positions themselves sideways in front of the marked line. Subsequently, the medicine ball is raised next to the head with a simultaneous bending of the knee, while the non-dominant hand is raised upwards to mimic the serve position. Following this, the medicine ball is thrown forward as far as possible without crossing the line or moving the legs. The distance is then measured from the starting line to the point of initial contact of the medicine ball in meters. Two repetitions of this exercise are carried out, and the best outcome is considered during data analysis. 

SMBT: The participant is positioned with their back pressed against the wall, their knees bent at a 90-degree angle, and their feet resting flat on the floor. He holds a medicine ball weighing 3 kg at shoulder level. The objective is to throw the medicine ball as far forward as possible while maintaining contact between the head, shoulders, and back and the wall. Meanwhile, the non-dominant hand remains on the abdomen. The distance is measured from the wall to the point where the medicine ball initially makes contact with the ground. This test is conducted three times, and the average of the results is utilized for analysis.

GS: This assessment is conducted solely with the dominant arm, which is fully extended at the elbow and aligned with the body. The participant is instructed to exert maximum voluntary force by tightly gripping the dynamometer for a duration of three seconds. The force exerted during each handshake is measured in kilograms (kg) using a specialized instrument. A total of three handshakes are performed, and the result is determined by calculating the average value of all the measurements obtained.

TS: The participant stands in an upright position, holding the bat with his left fist in front of a marked scale. Simultaneously, he embraces the baton with his right fist, aligning it with point zero. The task is imitated by lifting the bat in front of the participant while separating the arms. As the right fist slides along the bat, the left fist remains stationary. The objective is to execute an overhead spark with fully extended arms, aiming to minimize the distance between them. This sequence is repeated three times consecutively, without any breaks in between. The outcome is measured in centimeters, with the best result being recorded as the smallest distance between the hands during the spark.

SS: The speed of the first service is measured using a radar gun. Positioned four meters behind the player, the radar is aimed towards the court’s center. A total of twelve straight serves are executed on each side of the court (in the “deuce” and “advantage” serve boxes), with a 60 s break between the two series. It is crucial that a flat serve technique is employed, and the speed of the ball is measured in kilometers per hour. Only serves that land within the serve boxes are taken into account. Average serve values are documented for data analysis.

### 2.4. Experiemental Protocol

The research was conducted in two stages: (1) evaluating the participants’ motor skills and (2) assessing the speed of the serve. All measurements were taken within a single day. Before the testing, all participants engaged in a standard warm-up routine for tennis training. Additionally, before the serve speed assessment, all participants followed a specific warm-up protocol consisting of 5 min of dynamic shoulder movements and 20 slow serves. Tests for the assessment of explosive strength of the throwing type, a test of the strength of the hand and forearm muscles, and a test for assessing the flexibility of the arms and shoulder girdle were performed immediately after warming up, while the measurement of the serve speed was carried out at the very end. The tests that followed were conducted in the order described (OMBT, BMBT, MBTSP, SMBT, GS, TS), followed by speed of serve (SS). The recovery time between each test had to be two minutes. To mitigate the impact of weather conditions, all tests were conducted on an indoor hard tennis court.

### 2.5. Statistical Analysis

Statistical analysis was performed with the use of Statistica 14.0.1.25 (TIBCO Software, Inc., Palo Alto, CA, USA). The level of statistical significance was set at *p* < 0.05. Basic descriptive parameters (mean—x; standard deviation—SD) were used to describe each variable. The association between quantitative variables was determined using the Pearson correlation coefficient (r). Multiple linear regression was also used to identify factors that predict service speed. The average serve speed was used as a dependent variable in the analysis of multiple regression, while all other variables (OMBT, BMBT, MBTSP, SMBT, GS and TS) were used as independent predictors.

## 3. Results

The basic descriptive indicators (Mean, Min, Max, and St. Dev.) and the correlation of OMBT, BMBT, MBTSP, SMBT, GS, and TS with SS were calculated, as shown in Table 1.

In the case of variables OMBT, BMBT, MBTSP, SMBT, and GS, the outcomes closely align with the average, and exhibit minimal variability. This suggests that there is little variation among participants in this particular aspect. Conversely, variables TS and SS display the highest dispersion of results.

Table 1 presents the correlation between the observed variables and the serve speed. The correlation between OMBT and SS, MBTSP and SS, and GS and SS is statistically significant (*p* < 0.05). The correlation between BMBT and SS, SMBT and SS, and TS and (SS) is not statistically significant.

Based on the findings of the regression analysis presented in Table 2, it can be inferred that there is a statistically significant correlation of the explosive strength of the throwing type, the grip strength, and the flexibility of the arms and shoulder girdle with the speed of serve in female tennis players. The coefficient of multiple correlation is determined to be 0.97, while the coefficient of significance is reported as *p* < 0.05. Furthermore, the coefficient of determination, which stands at 0.83, suggests that approximately 83% of the variability in serve speed can be accounted for by the combination of independent variables under examination (explosive strength of the throwing type, grip strength and arms and shoulder girdle flexibility). In other words, the common influence of all these independent variables explains 83% of the observed variance.

Table 3 presents a linear regression model consisting of six steps, wherein an extra predictive variable is added in each step. The significance of MBTSP and GS is demonstrated in the first two steps. The MBTSP accounts for 51% of the variation in serve speed, while the combination of MBTSP and GS explains 58% of the serve speed variance.

The predicted variance after the third step suggests that at least 75% of the variance of the serve speed can be explained by MBTSP, GS and OMBT. The predicted variance after the fourth step suggests that at least 83% of the variance of the serve speed can be explained by MBTSP, GS, OMBT and SMBT. The predicted variance after the fifth step suggests that at least 87% of the variance of the serve speed can be explained by MBTSP, GS, OMB, SMBT and TS. The final prediction model consists of MBTSP, GS, OMBT, SMBT, TS and BMBT. These variables predict 83% of the serve speed variance.

## 4. Discussion

The presented results indicate that the overhead medicine ball throw, medicine ball throw shot put, and grip strength test exhibited a statistically significant correlation with serve speed among young tennis players.

A very high correlation (r = 0.75) was found between the MBTSP variable and the SS when analyzing all female tennis players. Furthermore, in the multiple linear regression analysis, MBTSP emerged as the primary variable in the predictive model, which explains the 51% variance of the serve speed. This means that 51% of the variance of serve speed can be predicted based on the results of the MBTSP. The examination of serve and MBTSP reveals that the actions performed during the active phase, such as extending the legs, arms, and wrists, play a substantial role in determining the speed of the serve. These movements account for a significant portion of the variability, ranging from 35% to 55%. 

This discovery is consistent with the results of a prior study [20]. Another similar study conducted on male players (aged 19.66 ± 1.63 years) suggested that 87% of the serve speed could be explained by the results of the MBTSP, further supporting its similarity to the serving motion [19]. Variable OMBT also showed a high correlation (r = 0.70). On the other hand, the results of the variable SMBT also showed a high correlation (r = 0.62), but it was statistically insignificant. The variable BMBT showed a medium correlation (0.43), which was also statistically insignificant. In terms of assessing upper body strength, OMBT is commonly used as an indicator of serve speed. Previous research [5,16,21] supports the relationship between throwing medicine balls and serve speed, particularly in male players. Based on the findings of this study, the aforementioned statement can be asserted regarding the examined group of young female tennis players. In a recent study [10], researchers found that the SMBT test showed a positive correlation between both the dominant and non-dominant hand and serve speed in 42 male competitive tennis players (23.9 ± 5.82 years). However, in contrast with this study, the results were deemed statistically insignificant. Despite the similarity in the need for a coordination of energy transfer through a kinetic chain, the throwing of the medicine ball from a sitting position and the performance of the serve may lead to different coordinations of body parts, resulting in a variety of outcomes. Identifying specific factors that can predict serve speed is essential in designing effective training programs, considering the significant impact of the serve on match outcomes. A well-executed serve relies on the efficient utilization of the kinetic chain, enabling optimal energy transfer upon impact. 

Consequently, the throwing of medicine balls is frequently used in teaching and training to enhance the upper body strength and facilitate the seamless connection of movements from the lower body. This approach enables the transfer of energy to the upper limbs, ultimately leading to the comprehensive physical conditioning necessary for a proficient serve. Previous research [22] highlights the issues related to throwing medicine balls with the aim of enhancing golf performance. The main emphasis lies in performing a throw that imitates the motion of a golf swing, incorporating factors like precise ball trajectory, high velocity, and considerable distance. Similarly, in tennis, when using medicine balls for tests that simulate specific shots, it is crucial to consider several of the aforementioned factors so as to closely replicate the performance elements in situational conditions.

Comparing tennis with various other sports such as golf, handball, or baseball, where exercises with medicine balls are often used to improve the overall quality of the shot and to assess the relationship with shot speed, in various research similar results can be observed. A study [23] conducted on baseball consisting of 18 female and 10 male participants revealed that the inclusion of a medicine ball during training sessions significantly increases the chances of achieving a higher shot speed, compared to the use of elastic bands as a tool for enhancing shot performance in terms of speed. In a previous study [24] conducted on young female handball players, with an average age in the experimental group of 11.23 ± 0.41 years and in the control group of 10.99 ± 0.4 years, using a medicine ball in training for 16 weeks, the findings revealed significant improvements in tests of throwing a medicine ball with one hand, which can ultimately lead to a more powerful and faster shot.

In a previous study [25], a group of tennis players with an average age of 14.66 ± 1.98 years took part in research that examined two different types of medicine ball throws (MBT): forward and backward. The findings of the study reveal that the backward medicine ball throw was a significant predictor of serve speed. MBT has a distinct correlation with a tennis serve, as players must understand the kinetic chain of their entire body. This understanding enables them to effectively transfer power and achieve optimal performance. It is important to note that while there is a strong correlation between the speed of the first serve and MBT (backward), this does not automatically guarantee an enhancement in serve speed. Nevertheless, if training is focused on enhancing tennis shots and building physical strength concurrently, this approach could prove beneficial in improving the aforementioned skills. Furthermore, in a previous study [26], it was found that MTB tests were strong indicators of serve speed in male tennis players under the ages of 13 and 15. Additionally, medicine ball throwing on the forehand side (MBF) in the under 13 category and OMBT in the under 15 category, along with body weight, amongst female players were significantly correlated with serving speed. These findings suggest that the disparities between male and female players start to emerge during the early stages of puberty. These results also align with previous studies [5], which have emphasized the significance of body mass in the serving speed of female players, particularly in younger players in the under 14 and under 18 categories. The upper body measurements, such as MBF, explained 10–40% of the serve variance for both male and female players. Therefore, it appears that upper body strength and the ability to transfer power from the lower to the upper body, including rotational movements, play a crucial role in achieving optimal results in serve speed. The findings align with previous research conducted on young athletes and emphasize the significance of upper body strength and serve performance, while suggesting that anthropometric variables have a lesser influence [5,27]. In addition, in a previous study [5], which involved 1019 male and female tennis players aged 12 to 18, similar outcomes were observed. The results indicate that MBT, grip strength, body height, and body mass are correlated with serve speed. Furthermore, the analysis has revealed that a combination of various factors (MBT, grip strength, and body weight) accounts for 41–66% of the variability in serve speed among boys and 19–45% among girls. Regarding the impact of selected predictors on serve speed across all groups, it was highlighted that upper body strength, as assessed through medicine ball throw and grip strength, had the most significant influence. 

Grip strength is a widely used measure of strength in younger players, serving as an indicator of overall strength development in boys and girls during childhood. Therefore, these research results underscore the importance of grip strength in predicting serve speed. A correlation between grip strength and serve speed in national groups of players was found in a study [19], however, no such correlation was observed among professional-level players. The wrist’s contribution to the kinetic chain is crucial, as it transmits the final speed of the ball without directly generating strength. This observation may explain the absence of an apparent link between grip strength and serve speed in professional players, indicating a potentially more efficient kinetic chain compared to players at lower levels. In one study [5], the strength of the grip correlated positively with the speed of shots in younger players, although the correlation was stronger in male than in female tennis players. In the same paper, it was interesting to note that body mass, again in female players aged 14–18 years, seemed to be a key anthropometric factor, which was confirmed by the results of regression analysis. Due to the benefits associated with force production, higher body mass seems to be tolerated in women’s tennis, given the potentially positive effects on stronger shot production.

The significance of the serve speed in tennis is just as crucial as the speed of shots from the baseline. Particularly in younger players, technical abilities are paramount for achieving success in the game, indicating a strong correlation between the performance of the serve and the player’s technical qualities at that age [28]. Bearing this in mind, the primary emphasis during this training period will be on the overall technical advancement of the player. However, as the player progresses and reaches the later stages of development, the importance of physical attributes and improved fitness become increasingly vital, as they greatly contribute to generating powerful shots. 

Furthermore, apart from possessing strength and power, the range of motion of players can also impact their movements. Previous research [29] has indicated a positive relationship between the flexibility of the shoulder, elbow, and wrist joints and the speed of the tennis serve. A wider range of motion in these joints enables greater momentum of the racket, resulting in a higher generated force and a faster serve. Maintaining adequate flexibility ensures the anatomical range of motion in the joints and optimal muscle length at rest, enhances muscle flexibility, improves the coordination of movements, and ultimately enhances the effectiveness of force application [30]. No significant correlation was found between serve speed and the test measuring hand and shoulder girdle flexibility in this study. However, a previous study [31] conducted on tennis players of a similar age group (14.30 ± 2.22 years old) but different sexes revealed a connection between shoulder joint flexibility and serve speed. It is worth noting that shoulder joint flexibility tends to decrease with age, particularly among younger tennis players who experience repetitive and one-sided upper body loads inherent to this sport. Consequently, the development of the upper body, particularly the shoulder joint, is a crucial aspect of the training regime for young tennis players. In previous research [20], it was found that female tennis players aged 13 to 14 highlighted the significance of having optimal flexibility in the shoulder girdle. This flexibility was measured using a stick and was observed to have a direct impact on their serve and smash techniques. By enabling faster and smoother movements, it played a crucial role in preparing for and executing these shots effectively. Women tend to have greater flexibility compared to men, which can help alleviate the limited internal rotation of the glenohumeral joint in female athletes. Additionally, the lower impact strength of female players can contribute to reduced impact and damage to muscle structure and soft shoulder tissues, ultimately leading to decreased mobility of the glenohumeral joint [32]. Based on the aforementioned details regarding the significance of hand and shoulder girdle flexibility, it is recommended that tennis players focus on increasing this skill to enhance their serve speed. A correlation was discovered in a study [33] between the serve speed of female players below the age of 12 and their ranking position. It was found that players with higher rankings tend to deliver faster serves. By delivering faster serves, the server’s chances of success are heightened while simultaneously decreasing the opponent’s chances, although it must be mentioned that it is key that their movements align with the ball’s trajectory within a limited timeframe whilst attempting to serve. This presents a significant challenge for female players in this age group. During the initial stage of puberty, girls tend to exhibit more advanced physical maturation compared to boys. As a result, their physical abilities can have a greater influence on their competitive performances. It is crucial, therefore, to begin training that takes into consideration the differences between the sexes at this stage. In light of the growing significance of the serve in modern tennis, it becomes imperative to identify the primary predictors of serve speed to develop the most effective training strategies.

However, it is important to acknowledge the limitations of this study. Firstly, it would be beneficial to create a regression formula for each age category of players (12, 14, 16, 18 years) by involving a larger number of participants. Secondly, it would be advantageous to sort the sample based on their performance on the ranking list, and group them according to their results in the tested skills. Secondly, the use of a medicine ball of the same weight was applied for all throws recorded in this research, but it could be suggested that the tests used could be more heavily loaded according to the biomechanical factors of the throw. Future research could explore more comprehensive training programs and examine their impacts on strength, while also monitoring the correlation with long-term effects on serve speed. However, these findings provide valuable insights for coaches seeking to create specific fitness training regimes aimed at enhancing players’ motor skills, particularly in terms of serve speed. 

## 5. Conclusions

To summarize this paper, it can be emphasized that the predictive factors of serve speed are intricate and interconnected. The findings of this study indicate that the outcomes of upper body strength tests serve as reliable indicators for estimating serve speed in young female tennis players. The study discovered a notable correlation between the speed of serve and the results of explosive strength tests of the throwing type MBTSP, OMBT, as well as GS. By enhancing upper body strength, young female tennis players have the potential to improve their serve speed and subsequently their overall performance.

## Figures and Tables

**Table 1:** Basic descriptive indicators of observed variables and their correlation.

Variables	Mean	Min	Max	St. Dev.	R(X,Y)	*p*
OMBT	6.29	5.70	7.2	0.53	0.70	0.02 *
BMBT	7.24	5.80	9.10	1.15	0.43	0.21
MBTSP	7.07	6.00	8.35	0.80	0.75	0.01 *
SMBT	1.79	1.71	1.89	0.05	0.62	0.06
GS	28.67	25.64	32.30	1.91	0.71	0.02 *
TS	73.86	57.00	96.50	10.77	0.57	0.09
SS	108.07	89.56	125.65	12.12		

Legend: OMBT—overhead medicine ball throw; BMBT—backward overhead medicine ball throw; MBTSP—medicine ball throw shot put; SMBT—seated medicine ball throw; GS—grip strength; TS—twist with the stick; SS—serve speed; *—significant correlation (*p* < 0.05); Mean—average value; Min—minimum value; Max—maximum value; St. Dev.—standard deviation.

**Table 2:** Results of regression analysis.

	SS Beta	*p*-Value
OMBT	−0.04	0.89
BMBT	0.09	0.76
MBTSP	0.34	0.25
SMBT	0.25	0.46
GS	0.41	0.22
TS	0.41	0.32
R	0.97	<0.05
Rsq	0.83	<0.05

Legend: SS—serve speed; OMBT—overhead medicine ball throw; BMBT—backward overhead medicine ball throw; MBTSP—medicine ball throw shot put; SMBT—seated medicine ball throw; GS—grip strength; TS—twist with the stick; R—coefficient of the multiple correlation; Rsq—coefficient of the determination.

**Table 3:** Predictive model.

MODEL	R	Rsq	Adjusted Rsq	Standard Error of the Estimate
MBTSP	0.75	0.57	0.51	8.48
MBTSP + GS	0.82	0.67	0.58	7.89
MBTSP + GS + OMBT	0.91	0.84	0.75	6.02
MBTSP + GS + OMBT + SMBT	0.95	0.91	0.83	4.93
MBTSP + GS + OMBT + SMBT + TS	0.97	0.94	0.87	4.37
MBTSP + GS + OMBT + SMBT + TS + BMBT	0.97	0.94	0.83	4.96

Legend: MBTSP—medicine ball throw shot put; GS—grip strength; OMBT—overhead medicine ball throw; SMBT—seated medicine ball throw; TS—twist with the stick; BMBT—backward overhead medicine ball throw; R—coefficient of the multiple correlation; Rsq—coefficient of the determination.

## Data Availability

Data available on request.
